# Hairy cell leukemia presenting with progressive pericarditis and pleuritis

**DOI:** 10.1007/s00277-018-3378-6

**Published:** 2018-05-30

**Authors:** Atsushi Iwashige, Makoto Hirosawa, Junichi Tsukada

**Affiliations:** 1 Hematology, University of Occupational and Environmental Health, 1-1 Iseigaoka, Yahatanishi, Kitakyushu, 807-8556 Japan; 2 Palliative Care Center, University of Occupational and Environmental Health, Kitakyushu, Japan

Dear Editor,

Hairy cell leukemia (HCL) has been recognized as an indolent B cell leukemia characterized by splenomegaly, pancytopenia, and neoplastic cells morphologically with irregular cytoplasmic hair-like projections on smears [1]. However, body fluid retention, such as ascites and pleural effusion, are extremely rare in HCL [2–4].

**Fig. 1:**
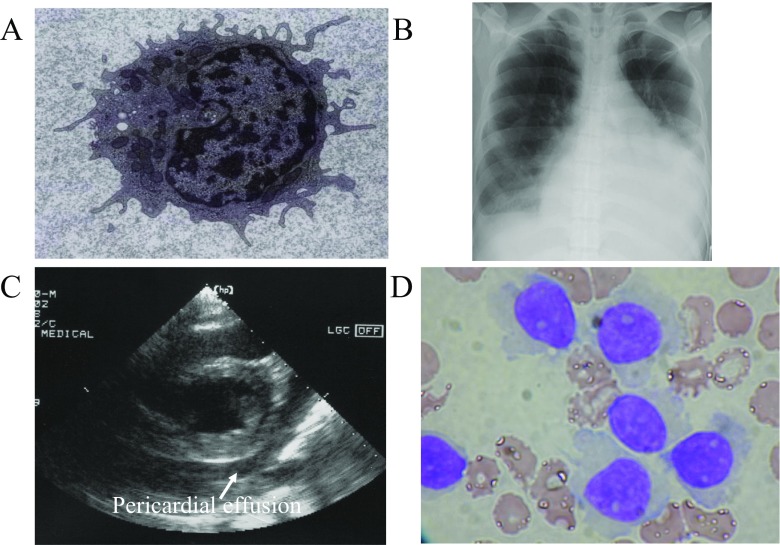
Transmission electron microscopy demonstrated hairy cytoplasmic projections along the cellular border of peripheral blood atypical lymphoid cells (**a**). Chest X-ray showed pleural effusion and cardiomegaly (**b**). Echocardiography examination exhibited pericardial effusion (**c**). Atypical lymphocytes with hairy cytoplasmic projections were observed in the pericardial effusion (**d**; Wright-Giemsa staining × 400)

A 43-year-old Japanese female was referred to our hospital because of a 2-month history of fever and general fatigue. She had suffered from appetite loss and abdominal fullness for 2 years before admission. Physical examination revealed anemia and palpable spleen. Lymph nodes were not palpable. Blood examination showed leukocyte count of 3.5 × 10^9^/L with 81% atypical lymphocytes. Soluble IL-2 receptor was 43,974 U/mL. The atypical lymphocytes were medium-sized with hair-like cytoplasmic projections and were positive for CD19, CD20, CD22, CD11c, CD25, FMC7, and CD103. Images obtained from phase contrast microscopy and transmission electron microscopy (Fig. 1a) also showed hairy cytoplasmic projections along the cellular border. The fried-egg appearance with diffuse infiltration of atypical cells was observed on bone marrow biopsy.

Inflammatory conditions have continued with high fever and serum C-reactive protein levels around 20 mg/dL. Chest X-ray showed pleural effusion and cardiomegaly (Fig. 1b). Echocardiography exhibited pericardial effusion (Fig. 1c). Since dyspnea and hypotension were observed on day 8 after admission, the pericardial and pleural effusions were drained. The effusions were exudative with cell infiltration consisting of atypical lymphocytes (Fig. 1d) and neutrophils. Atypical lymphocytes in the effusions were also positive for CD19, CD20, CD22, CD11c, CD25, FMC7, and CD103. Extensive laboratory testing for autoimmune diseases, bacteria, virus, fungus, and tubercle bacillus were all negative.

IL-6 production has been demonstrated in HCL leukemic cells [5, 6]. In our reverse transcription-polymerase chain reaction analysis, her effusion HCL cells but not her peripheral blood HCL cells showed significant expression of IL-6 mRNA. A remarkably high level of IL-6 (2900 pg/mL) was also observed in the pericardial effusion, compared with that in serum (5.42 pg/mL). G-CSF levels were elevated in both serum (318 pg/mL) and the effusion (2530 pg/mL). Neither TNFα nor GM-CSF was elevated. Methylprednisolone administration improved her inflammatory conditions such as fever. The pericardial and pleural effusions were significantly decreased after the first cycle of pentostatin (5 mg/m^2^). However, after the first cycle, pentostatin was discontinued due to skin rash in her trunk and extremities, which were suspected to be caused by pentostatin. Therefore, she was treated with cladribine (0.09 mg/kg for 7 days), which completely resolved both pleuritis and pericarditis. There has been no recurrence of pleuropericarditis as well as HCL for 5 years.

To our knowledge, this is the first report of HCL presenting with progressive pericarditis. Early recognition and intervention prolong survival in patients with malignant pericardial effusion, especially in those with chemotherapy-sensitive malignancies such as lymphoma. In our case, an initial relief of the symptoms was obtained by effusion drainage. Further improvement was achieved with systemic chemotherapy. Elevated concentrations of IL-6 have been shown in pericardial effusion associated with hematological malignancies [7–9]. In our case, an extremely high IL-6 concentration was observed in the pericardial effusion. IL-6 mRNA expression was detected only in the effusion HCL cells, suggesting that effusion IL-6 might be involved in the fluid retention and systemic inflammatory responses.

Because the patient has died of breast cancer brain metastasis and her kin are not traceable, the ethics committee of our institute has approved publication of this report.
